# L-Ascorbic Acid 2-Phosphate Attenuates Methylmercury-Induced Apoptosis by Inhibiting Reactive Oxygen Species Accumulation and DNA Damage in Human SH-SY5Y Cells

**DOI:** 10.3390/toxics11020144

**Published:** 2023-02-02

**Authors:** Kuiyang Zuo, Qi Xu, Yujie Wang, Yutong Sui, Ye Niu, Zinan Liu, Mingsheng Liu, Xinpeng Liu, Dan Liu, Wei Sun, Ziyu Wang, Xiaomei Liu, Jinyu Liu

**Affiliations:** Department of Toxicology, School of Public Health, Jilin University, 1163 Xinmin Avenue, Changchun 130021, China

**Keywords:** methylmercury, reactive oxygen species, DNA damage, DNA repair, apoptosis, AA2P, vitamin C

## Abstract

Methylmercury (MeHg) is a toxin that causes severe neuronal oxidative damage. As vitamin C is an antioxidant well-known to protect neurons from oxidative damage, our goal was to elucidate its protective mechanism against MeHg-induced oxidative stress in human neuroblastomas (SHSY5Y). We treated cells with MeHg, L-ascorbic acid 2-phosphate (AA2P), or both, and used MTT, flow cytometry, and Western blot analyses to assess cell damage. We found that MeHg significantly decreased the survival rate of SH-SY5Y cells in a time- and dose-dependent manner, increased apoptosis, downregulated PAR and PARP1 expression, and upregulated AIF, Cyto C, and cleaved Caspase-3 expression. A time course study showed that MeHg increased reactive oxygen species (ROS) accumulation; enhanced apoptosis; increased DNA damage; upregulated expression ofγH2A.X, KU70, 67 and 57 kDa AIF, CytoC, and cleaved Caspase-3; and downregulated expression of 116 kDa PARP1, PAR, BRAC1, and Rad51. Supplementation with AA2P significantly increased cell viability and decreased intrinsic ROS accumulation. It also reduced ROS accumulation in cells treated with MeHg and decreased MeHg-induced apoptosis. Furthermore, AA2P conversely regulated gene expression compared to MeHg. Collectively, we demonstrate that AA2P attenuates MeHg-induced apoptosis by alleviating ROS-mediated DNA damage and is a potential treatment for MeHg neurotoxicity.

## 1. Introduction

MeHg is an organic heavy metal pollutant that can pass through the blood–brain barrier [1] and cause damage to the central nervous system (CNS), making it a target organ for MeHg toxicity [2,3]. As such, studies have found that MeHg can lead to a variety of neurological complications, including increased intracellular reactive oxygen species (ROS) levels while reducing the expression of anti-apoptotic Bcl-2 protein in the PC12 cell line derived from the adrenal medulla of rats [4]. MeHg also causes hippocampal damage, learning and memory impairment [5], and neurobehavioral disorders [6]. The neurotoxic effects of MeHg on the developing CNS may be related to its prooxidative properties [7]. Both low- and high-dose MeHg exposure results in impaired brain antioxidant capacity [8]. Mice exposed to MeHg (4 mg/kg) for 15 days were shown to have increased mitochondrial damage and memory impairment, and neuronal cell death [9]. It was also shown that methylmercury can damage hippocampal neurons. In rats injected with 2 mg/kg of MeHg subcutaneously for 50 days, pathological tests confirmed that cognitive function was impaired and the ultrastructure of hippocampal tissue was altered [10]. Several studies have described the overproduction of ROS or reduced antioxidant defenses as some of the main causes of MeHg-related neurotoxicity [11,12,13]. At present, there are several mechanisms of MeHg-induced injury, in which ROS are an important factor [14]. MeHg can increase intracellular ROS [15,16] and cause lipid peroxidation, which leads to oxidative stress in cells [17] and exerts toxic effects [18]. Therefore, the activation of antioxidant mechanisms prevents MeHg poisoning [19].

Studies have shown that MeHg accumulation in mitochondria, to a certain extent, also causes oxidative damage [20], and ROS generated in cells due to MeHg exposure are amplified in mitochondria to induce further neuronal damage, mitochondrial transmembrane potential, and decrease oxidative phosphorylation enzyme activity [21]. These findings suggest that mitochondria play an important role in MeHg-induced neuronal damage [22]. The incidence of MeHg-mediated oxidative stress should depend on the ability of the intracellular redox system to respond to the consequences of interactions between the MeHg-induced events described above. Once MeHg-mediated oxidative stress occurs, it may trigger the activation of various cellular signaling pathways, leading to cellular damage.

Another study showed that MeHg causes DNA double-strand breaks (DSBs) [23]. In addition, MeHg was also shown to interact with DNA to form MeHg-DNA adducts or generate ROS-triggered oxidative damage [24]. Therefore, MeHg is believed to induce cell death owing to its genotoxicity and promotion of DNA damage [25]. MeHg can also induce neuronal apoptosis through the mitochondrial pathway; oxidative stress plays an important role in mediating the activation of the apoptosis pathway [26]. MeHg can affect mitochondrial function, inhibit mitochondrial complex activity (complex III and complex IV), reduce ATP production [27], change mitochondrial membrane potential [28], open the mitochondrial permeability transition pore, and increase mitochondrial permeability. Cytochrome C (Cyto C) is released into the cytoplasm [29] and activates the downstream apoptosis-executing protein caspase 3 (Caspase 3) to induce apoptosis [30,31] and increase lipid, protein, and DNA peroxidative damage [32]. MeHg induces apoptosis in cerebellar granule cells, Apoptosis-inducing factor (AIF) translocates from the mitochondria to the nucleus, and chromatin condensation and DNA degradation are detected [33]. Nuclear translocation of AIF is caspase-independent in MeHg-induced apoptosis of cerebellar granule cells [34]. In fact, a recent study showed that MeHg can damage brain cells by activating mitochondria-mediated caspase-dependent and -independent AIF-poly (ADP-ribose) polymerase (PARP1) apoptotic signaling pathways [35,36].

Vitamin C (VC), chemical name—ascorbic acid, is a hydrophilic molecule that can exist in reduced or oxidized forms [37]. VC is a potent antioxidant, and sodium l-ascorbic acid-2-phosphate (AA2P) is a stable VC derivative [38]. AA2P is absorbed by cells and converted to active ascorbic acid by various enzymes. VC is an indispensable antioxidant and a cofactor for the optimal function and development of eukaryotic cells. VC was shown to inhibit the production of free radicals and alleviate oxidative damage caused by ROS [39]. In addition to being a powerful antioxidant, VC also helps by protecting key macromolecules such as proteins, lipids, and DNA from oxidation. By maintaining important tissue structure and function [40], VC can also improve the survival rate of nerve cells [41]; therefore, it has long been used in pharmaceutical preparations. In the brain, VC is involved in multiple processes, including the synthesis of catechol, amino acids, and cholesterol in addition to its general antioxidant activity [42]. VC can also directly remove ROS or reactive nitrogen generated during brain metabolism. For example, adding VC to cells cultured in vitro can prevent lipid peroxidation [43]. In vitro studies have also shown that VC promotes the differentiation of embryonic stem cells into neurons [44]. In addition, VC is required to improve learning and memory abilities [45]. Studies have shown that VC can significantly reduce MeHg-induced oxidative stress and has a protective effect on the nervous system [46]. VC reduces MeHg-induced lipid peroxidation and prolongs survival in mice [47]. VC can modulate the response to oxidative stress and DNA damage by altering redox signaling. VC post-treatment was observed to reduce the frequency of chromosomal aberrations by approximately 30% and significantly reduce the number of DNA breaks, which according to the authors, indicates the impact on DNA repair processes [48]. VC (100 and 500 mM) protects the epidermal surface both by stimulating collagen synthesis as well as inhibiting oxidative stress, apoptosis, and reducing the frequency of DNA damage [49]. VC also protects against MeHg-induced cell death in primary cultures of cortical neurons [50].

In this study, we aimed to investigate MeHg-induced injuries in human neuroblastoma-derived SH-SY5Y cells and to elucidate the mechanism behind the neuroprotective effects of AA2P against MeHg in the CNS. To accomplish this, we investigated ROS production, DNA damage, and apoptosis in cells exposed to MeHg to determine whether AA2P reduces cell damage and apoptosis by inhibiting MeHg-induced ROS. Furthermore, we identified the relationship between MeHg-induced ROS production, DNA damage, and apoptosis in AA2P-treated SH-SY5Y cells. Our work demonstrates that AA2P plays an important role in preventing MeHg-induced SH-SY5Y cell death and is a potential drug for the treatment of MeHg neurotoxicity.

## 2. Materials and Methods

### 2.1. SH-SY5Y Cell Culture

SH-SY5Y cells were purchased from the Cell Bank of Wuhan University, China Type Culture Collection, and certified by STR. SH-SY5Y cells were cultured in Dulbecco’s modified Eagle’s Medium (DMEM)/Nutrient mixture F-12 (F12) (Gibco, Grand Island, NY, USA) containing 10% fetal bovine serum (FBS; Hyclone, Logan, UT, USA). Cells were cultured at 37 °C and 5% CO_2_. When the cells proliferated to 80–90% confluence, they were digested and subcultured under the same individual conditions.

### 2.2. Treatment of SH-SY5Y Cells with MeHg and AA2P

When SH-SY5Y cells proliferated to 70–80% confluence in DMEM/F12 medium containing 10% FBS, SH-SY5Y cells were first treated with 0.5–2 μM MeHg prepared in DMEM/F12 medium without FBS for 24 h. Second, SH-SY5Y cells were treated with 1 μM MeHg for 0–24 h. For 2-Phospho-L-ascorbic acid trisodium sait (AA2P, Millipore SIGMA, Burlington, MA, USA, Lot:BCBM4646V), SH-SY5Y cells were first prepared with 100–500 μM AA2P in 1× PBS and treated with 1 μM MeHg for 24 h. For observation of cell survival, SH-SY5Y cells were treated with 300 μM AA2P and/or 1 μ M MeHg for 3 h, 12 h, and 24 h to observe intracellular reactive oxygen species production and related protein expression in SH-SY5Y cells.

### 2.3. Reactive Oxygen Species Assay

To detect ROS, 6.25 × 10^5^/well of SH-SY5Y cells were spread on 6-well plates, and SH-SY5Y cells were treated with 1 μM MeHg for 0–24 h when SH-SY5Y cells proliferated to 70–80% confluence. For AA2P, SH-SY5Y cells were treated with 300 μM AA2P and/or 1 μM MeHg for 3 h, 12 h, and 24 h. The supernatant of treated SH-SY5Y cells was collected; cells were digested with 0.25% EDTA-free trypsin for 1 min; digestion was terminated with DMEM/F12 medium containing 10% FBS; cells were washed twice with 1× pre-chilled PBS, resuspended in FBS-free medium, and centrifuged at 300× *g* for 5 min. The supernatant was discarded and the cells were then treated with a 5 μM DCFH-DA probe and incubated at 37 °C for 30 min in the dark. Cells were washed twice with pre-cooled PBS, resuspended in 400 μL of FBS-free medium, detected by flow cytometry (BD, Franklin, NJ, USA), and analyzed using FlowJo software.

### 2.4. Apoptosis Assays

To detect apoptosis, 6.25 × 10^5^/well of SH-SY5Y cells were spread on 6-well plates and SH-SY5Y cells were treated with 0.5–2 μM MeHg for 24 h or with 1 μM MeHg for 0–24 h when SH-SY5Y cells proliferated to 70–80% confluence. For AA2P, SH-SY5Y cells were treated with 300 μM AA2P and/or 1 μM MeHg for 12 h and 24 h. The supernatant of treated SH-SY5Y cells was collected and the cells were digested with 0.25% EDTA-free trypsin for 1 min. After digestion, the digestion was terminated with DMEM/F12 medium containing 10% FBS, the cells were washed with 1× pre-cooled PBS and cell counting was performed to ensure that the number of cells per group was approximately 5 × 10^5^ cells per group. Then, 5 × 10^5^ SH-SY5Y cells were suspended in 100 μL of binding buffer containing 5 μL Annexin V-FITC and incubated for 10 min at room temperature in the dark. After incubation, 5 μL PI was added and incubated for 10 min under the same conditions. SH-SY5Y cells were then analyzed by flow cytometry (BD, Franklin, NJ, USA).

### 2.5. Alkaline Comet Assay

An alkaline comet assay was performed on treated SH-SY5Y cells to detect damaged DNA. SH-SY5Y cells (1 × 10^5^/mL) were inoculated and cultured in a 6 cm dish to induce a logarithmic growth phase. SH-SY5Y cells were treated with 1 μM MeHg for 3 h, 6 h, 9 h, 12 h and 24 h, collected and counted, then cell suspensions were prepared with PBS. We ensured that each 100 μL of cell suspension contained 1 × 10^6^ cells. A glass slide was then covered with 90 μL of 1% normal melting point agarose, with excess agarose removed after cooling. Then, 80 μL of 0.5% low melting point agarose was mixed with 10 μL of the cell suspension, which was added dropwise onto the first layer of agarose. The cells were covered with a coverslip and kept on a flat surface. After cooling, the coverslip was removed and 60 μL of 0.5% low melting point agarose was added dropwise. The entire process was protected from light, and the prepared gel plate was lysed, untwisted, electrophoresed, and neutralized. The glue plate was removed to dry, and EB staining solution was added dropwise in the dark and left to stain the plate for 20 min prior to observation under a microscope for 2 h. Olive tail moment (OTM) was calculated as % tail DNA × distance from the center of the head to the center of the tail.

### 2.6. Western Blot Assay

Protein quantification was carried out using a Western blot assay. First, 4 × 10^6^ SHSY5Y cells were plated in a 100 mm cell culture dish and cultured in DMEM/F12 medium containing 10% FBS. When the cells reached 80% confluence, they were harvested and lysed in 200 μL RIPA buffer (Beyotime Biotechnology, Baoshan District, Shanghai, China) supplemented with 1% protease inhibitor cocktail (CoWin Biosciences, Hailing District, Taizhou City, China) and 1% phosphatase inhibitor cocktail (CoWin Biosciences, Hailing District, Taizhou City, Jiangsu Province, China) at 4 °C for 1 h. They were then centrifuged at 13,000× *g* for 20 min at 4 °C. The supernatant was collected, and protein concentration was analyzed using an Enhanced BCA Protein Assay Kit (Beyotime Biotechnology, Baoshan District, Shanghai, China). Then, 20 μg of protein per sample were loaded into each well and separated by 10% SDS polyacrylamide gel electrophoresis prior to being transferred to polyvinylidene difluoride membranes (Millipore, Massachusetts, USA). The membranes were incubated in 5% non-fat milk powder (Anchor, Auckland City, New Zealand) at room temperature for 1 h. The membranes were then incubated with the following primary antibodies: PARP1 rabbit mAb (1:1000; CST), AIF rabbit mAb (1:1000, CST), Cleave Caspase-3 rabbit mAb (1:1000, CST), γH2AX rabbit mAb (1:1000, CST), Cyt C rabbit mAb (1:1000; CST), KU70 rabbit mAb (1:1000; CST), BRCA1 rabbit mAb (1:1000; CST), RAD51 rabbit mAb (1:1000; CST), GAPDH mouse mAb (1:5000; ProteinTech Group Inc.), HRP-conjugated AffiniPure Goat Anti-Rabbit IgG (H + L) (1:10,000; ProteinTech Group Inc., Wuhan East Lake New Technology Development Zone, China), and HRP-conjugated AffiniPure Goat Anti-Mouse IgG (H + L) (1:10,000; Protein-Tech Group Inc., Wuhan East Lake New Technology Development Zone, China). Proteins were visualized using a chemiluminescence imaging analysis system (ECL; Tanon 5200; Shanghai Tianneng Technology Co., Ltd., Shanghai, China), and band intensity was analyzed using a Tanon-2500 fully automated digital gel image analysis system.

### 2.7. Statistical Analysis

GraphPad Prism version 8.0.2 (GraphPad Software, San Diego, CA, USA, 2018) and IBM SPSS 24.0 (IBM Corp., Armonk, NY, USA, 2016) were used for all analyses and preparations of graphs. All data represented in graphs were expressed as the mean ± SD. ANOVA and LSD methods were used to analyze significant differences; *p*-value was considered significant at *p* < 0.05.

## 3. Results

### 3.1. MeHg Decreases SH-SY5Y Cell Viability in a Time- and Dose-Dependent Manner and Increases Apoptosis in a Dose-Dependent Manner

**Figure 1:**
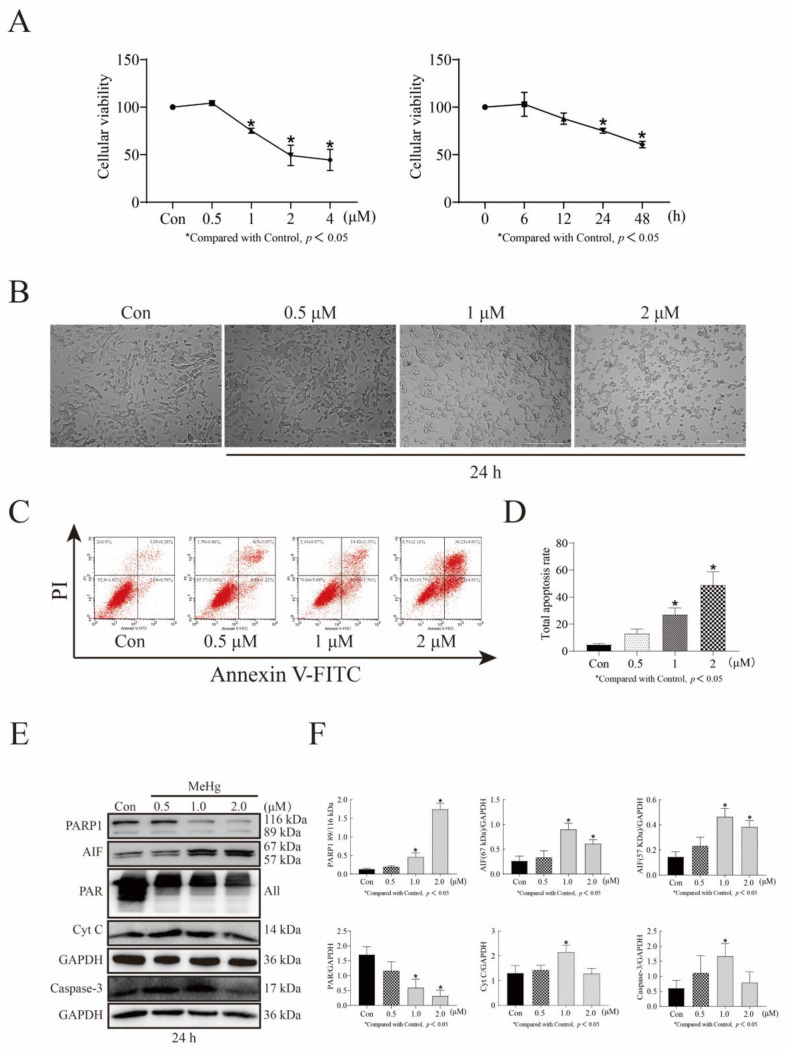
A cell damage model of MeHg was created by the treatment of SH-SY5Y cells with 0–4 μM MeHg at indicated time intervals. (**A**) MTT assay showing cell viability of SH-SY5Y cells treated with 0–4 μM MeHg for 6–48 h (**B**) The live cell workstation used to observe cell morphology of SH-SY5Y cells treated with 0–2 μM MeHg for 24 h. (**C**) Flow cytometry detection of apoptosis in 0–2 μM MeHg treated SH-SY5Y cells for 24 h. (**D**) GraphPad Prism 8.0.2 software was used to analyze the total apoptosis rate of SH-SY5Y cells treated with 0–2 μM MeHg for 24 h. Statistical significance was determined using ANOVA; *p* < 0.05. (**E**) Western blot analysis of the protein expression levels of PARP1, AIF, PAR, Cyt C, and cleaved Caspase-3 in SH-SY5Y cells treated with 0–2 μM MeHg for 24 h. (**F**) The Tanon-2500 fully automated digital gel image analysis system was used to analyze the net optical density of the Western blot strips. All values are represented as means ± SD (*n* = 3). Superscript denotes a statistically significant difference between groups (*p* < 0.05). Abbreviations: AIF, apoptosis-inducing factor; ANOVA, analysis of variance; Cyto C, cytochrome C; MeHg, methylmercury; MTT, 3-(4,5-dimethylthiazol-2-yl)-2,5-diphenyltetrazolium bromide; PAR, poly (adp-ribose); PARP1, poly (adp-ribose) polymerase 1; SH-SY5Y, neuroblastoma cells.

We first examined the cell survival rate of SH-SY5Y cells administered 0–4 μM MeHg for 6–48 h using an MTT assay and found that the cell survival rate decreased gradually with increasing time and concentration. The cell survival rates of SH-SY5Y cells treated with 0.5, 1, 2, and 4 μM MeHg for 24 h were 104.26%, 75.19%, 49.22%, and 44.44%, respectively (*p* < 0.05; Figure 1A). The survival rates of cells treated with 1 μM MeHg for 6, 12, 24, and 48 h were 103.02%, 87.95%, 75.19%, and 60.63%, respectively (*p* < 0.05; Figure 1A). Next, we treated SH-SY5Y cells with 0–2 μM MeHg for 24 h and observed through microscopy the changes in their morphology, which became more pronounced as concentration increased. The cells became rounder, the number of nuclei increased, the refractive index changed, and the number of viable cells gradually decreased (Figure 1B). Using flow cytometry, we also observed that the apoptosis rate gradually increased with increasing MeHg concentration and the percentage of apoptotic cells likewise increased. Notably, the total apoptosis rate of SH-SY5Y cells treated with 1 μM MeHg for 24 h was 24.4% (*p* < 0.05; Figure 1C,D). Using Western blot analysis to detect changes in the expression of the apoptosis indicators 116 kDa (uncleaved) PARP1 and Poly (ADP-Ribose) (PAR), we found that 1 μM MeHg treatment of SH-SY5Y cells for 24 h downregulated the expression of both. This same treatment upregulated the expression of 89 kDa PARP-1, 67 kDa AIF, 57 kDa AIF, 14 kDa Cyto C, and cleaved Caspase-3 (*p* < 0.05; Figure 1E,F). This suggests the presence of the PARP1/AIF apoptosis and Caspase-3 apoptosis pathways in MeHg-induced apoptosis. Based on these results, treatment of SH-SY5Y cells with 1 μM MeHg for 24 h was selected as the treatment condition for our cell-damage model.

### 3.2. MeHg Increases ROS Accumulation in SH-SY5Y Cells in a Time-Dependent Manner and Is Accompanied by an Increase in Apoptosis

**Figure 2:**
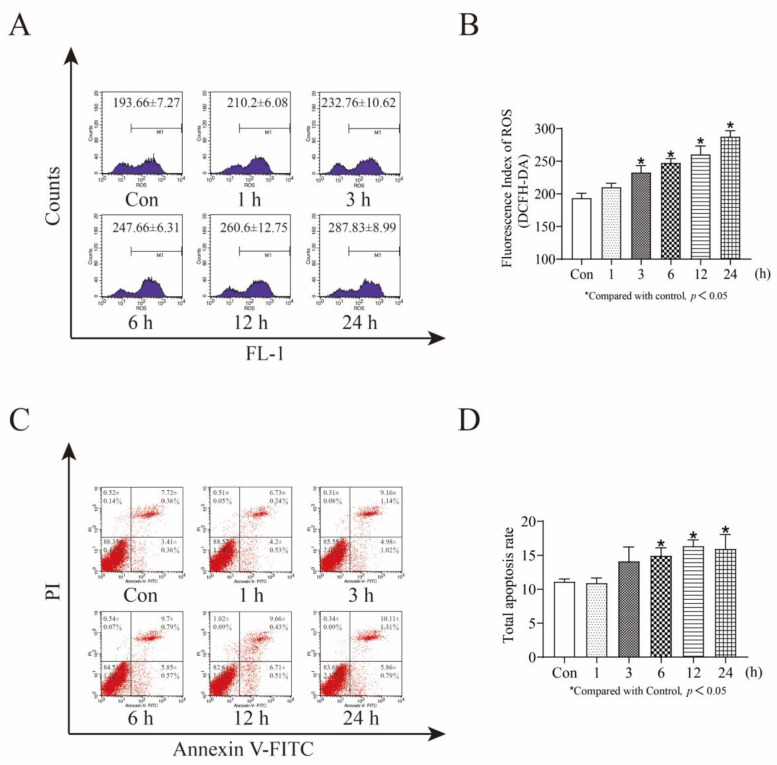
MeHg induces ROS accumulation and apoptosis in SH-SY5Y cells. (**A**) Flow cytometry detection of ROS levels in 1 μM MeHg-treated SH-SY5Y cells for 1–24 h. (**B**) GraphPad Prism 8.0.2 software analyses of ROS levels. (**C**) Flow cytometry detection of apoptosis in 1 μM MeHg-treated SH-SY5Y cells from 0–24 h. (**D**) Total apoptosis rate. Statistical significance was determined using ANOVA, *p* < 0.05. Abbreviations: DCFH-DA, ROS green fluorescent probe; ROS, reactive oxygen species.

First, we explored whether MeHg increases ROS accumulation in SH-SY5Y cells. Flow cytometric analysis showed that intracellular ROS increased significantly in a time-dependent manner following exposure to 1 μM MeHg for 1–24 h. The mean ROS at 3, 6, 12, and 24 h increased from 193.66 to 232.76, 247.66, 260.6, and 287.83, respectively (*p* < 0.05; Figure 2A,B). Second, to better demonstrate that MeHg induces apoptosis in SH-SY5Y cells, we examined the apoptosis of 1 μM MeHg-treated SH-SY5Y cells at 1, 3, 6, 12, and 24 h using flow cytometry. The total apoptosis rate increased from 11.13% to 15.97% as time increased (6 h: 14.95%, 12 h: 16.37%, 24 h: 15.97%, *p* < 0.05; Figure 2C,D).

### 3.3. MeHg Induces DNA-Damage-Mediated Repair and Caspase-Dependent and -Independent Apoptosis in SH-SY5Y Cells

**Figure 3:**
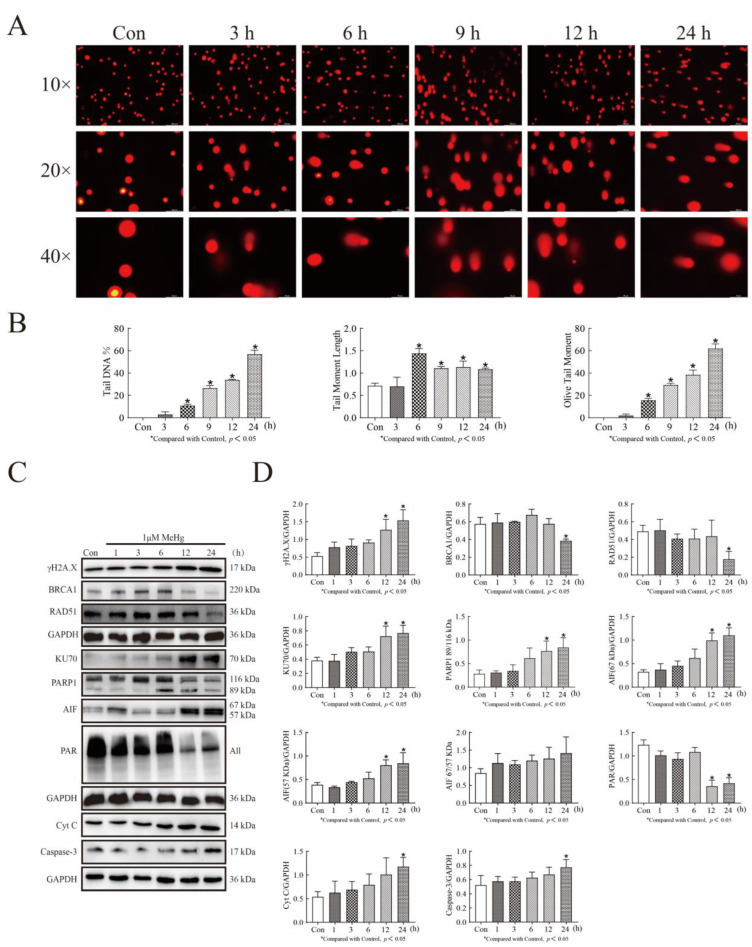
MeHg induces DNA damage and participates in both NHEJ repair and induced apoptosis in SH-SY5Y cells (**A**) The alkaline comet assay detects cell trailing in 1 μM MeHg-treated SH-SY5Y cells from 0–24 h. The images were photographed with a Leica fluorescence microscope. (**B**) CASP software analyses of comet image and the Olive tail moment can be used to determine the extent of DNA damage. (**C**) Western blot analysis detected the protein expression levels of γH2AX, BRCA1, RAD51, KU70, PARP1, AIF, PAR, Cyt C, and cleaved Caspase-3 in SH-SY5Y cells treated with 1 μM MeHg from 0–24 h. (**D**) The Tanon-2500 fully automated digital gel image analysis system was used to analyze the net optical density of the Western blot strips. The mean was calculated using GraphPad Prism 8.0.2 software. The data shown represent the mean from one of three independent experiments. Statistical significance was determined using variance (ANOVA). All values are represented as means ± SD (*n* = 3). Superscript denotes a statistically significant difference between groups (*p* < 0.05). Abbreviations: BRCA1 and RAD51, homologous repair-associated repair proteins; HR, homologous recombination; KU70, non-homologous end joining-associated repair protein; NHEJ, nonhomologous end joining; γH2AX, phosphorylated histone H2AX.

To observe the effect of MeHg treatment on DNA integrity in SH-SY5Y cells, we first examined potential DNA damage in 1 μM MeHg-treated SH-SY5Y for 1–24 h using an alkaline comet assay. We found that the total tail DNA content increased with increasing time, the head center to tail center distance increased, and the Olive tail moment increased, with statistically significant differences for all measurements at 6, 9, 12, and 24 h (*p* < 0.05, compared to control; Figure 3A,B). Furthermore, the expression of γH2A.X in SH-SY5Y cells treated with 1 μM MeHg from 1 to 24 h was detected by Western blot analysis, with MeHg significantly upregulating its expression (12 h: *p* < 0.05, 24 h: *p* < 0.05; Figure 3C,D). To demonstrate whether MeHg treatment affects proteins involved in DNA repair, we used Western blot analysis to detect the expression of proteins related to the homologous repair (HR) and non-homologous end joining (NHEJ) repair pathways. We found that 1 μM MeHg treatment of SH-SY5Y cells for 24 h downregulated the expression of HR repair pathway-related proteins BRCA1 and RAD51 and upregulated the expression of NHEJ repair pathway-related protein KU70 (*p* < 0.05; Figure 3C,D). To further investigate the role of the mitochondria-mediated apoptotic mechanism in MeHg-induced apoptosis in SH-SY5Y cells, we selected 1 μM MeHg treatment for 0–24 h. At 24 h, MeHg downregulated the expression of 116 kDa PARP-1 and PAR and upregulated the expression of 89 kDa PARP-1, 67 kDa AIF, 57 kDa AIF, Cyto C, and Caspase-3 (*p* < 0.05; Figure 3C,D).

### 3.4. AA2P Decreases MeHg-Induced ROS Accumulation in SH-5YSY Cells in a Dose-Dependent Manner

**Figure 4:**
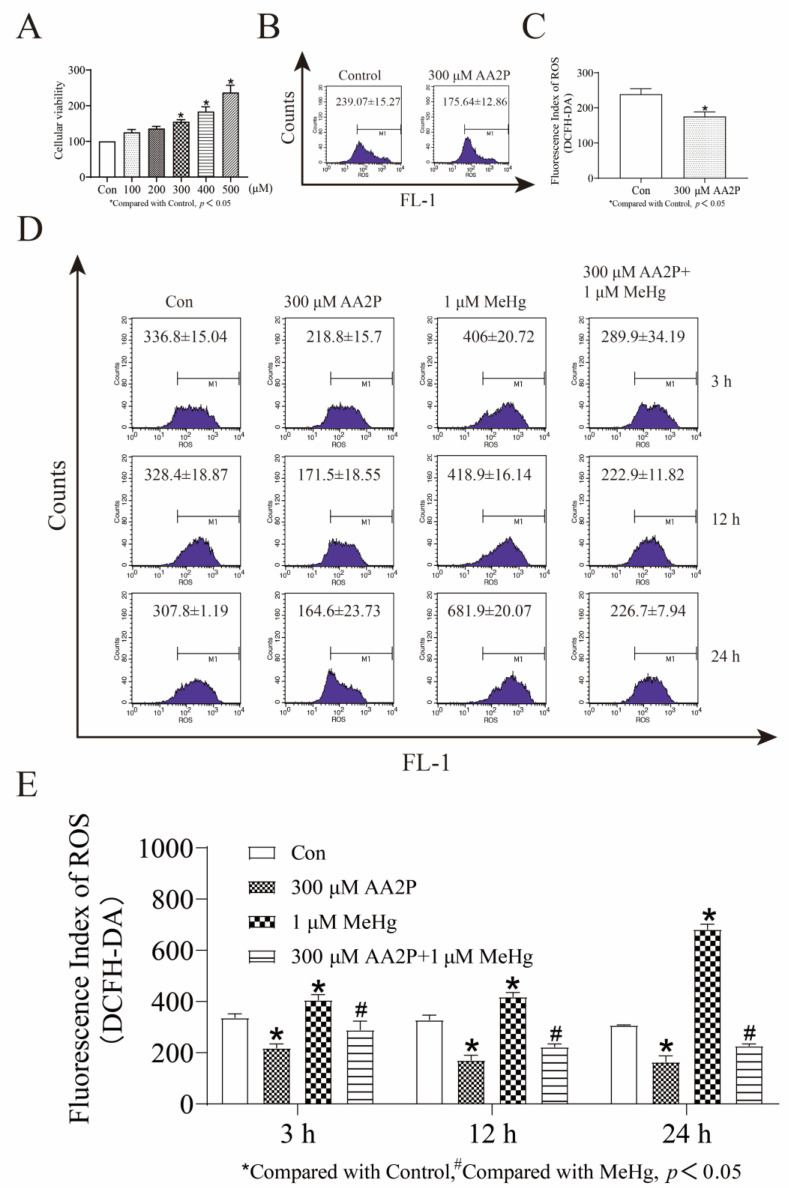
AA2P treatment increases the viability of SH-SY5Y cells and decreases intrinsic ROS levels and MeHg-induced ROS accumulation. (**A**) MTT assay for cell viability of 0–500 μM AA2P. (**B**) Flow cytometry detection of ROS levels in 300 μM AA2P-treated SH-SY5Y cells for 24 h. (**C**) GraphPad Prism 8.0.2 software analysis of ROS levels in 300 μM AA2P-treated SH-SY5Y cells for 24 h. (**D**) Flow cytometry detection of ROS levels in 300 μM AA2P- and 1 μM MeHg-co-treated SH-SY5Y cells for 3, 12, and 24 h. (**E**) GraphPad Prism 8.0.2 software analysis of ROS level in 300 μM AA2P- and 1 μM MeHg- co-treated SH-SY5Y cells for 3, 12, and 24 h. The mean was calculated using GraphPad Prism 8.0.2 software. The data shown represent the mean from one of three independent experiments. Statistical significance was determined using ANOVA. Abbreviations: AA2P, L-ascorbic acid-2-phosphate sodium salt.

To investigate the effect of AA2P treatment on MeHg-induced ROS production in SHSY5Y cells, we first examined the cell survival rate of SH-SY5Y cells treated with 0–500 μM AA2P for 24 h using an MTT assay. The survival rate of the 300 μM AA2P group was 155.15%, the 400 μM AA2P group was 183.75%, and the 500 μM AA2P group was 237.15% (*p* < 0.05; Figure 4A). We then used flow cytometry to verify the effect of AA2P on the ROS levels of treated cells. We found that 300 μM AA2P treatment for 24 h effectively reduced ROS accumulation (*p* < 0.05; Figure 4B,C). Finally, we selected 300 μM AA2P and 1 μM MeHg to co-treat cells and showed by flow cytometry that, first, 300 μM AA2P treatment alone of SH-SY5Y cells for 3, 12, and 24 h effectively reduced the cellular ROS equally and, second, that the AA2P and MeHg co-treatment of cells for 3, 12, and 24 h effectively reduced MeHg-induced intracellular ROS accumulation (*p* < 0.05; Figure 4D,E).

### 3.5. AA2P Decreases MeHg-Induced Apoptosis in SH-SY5Y Cells

**Figure 5:**
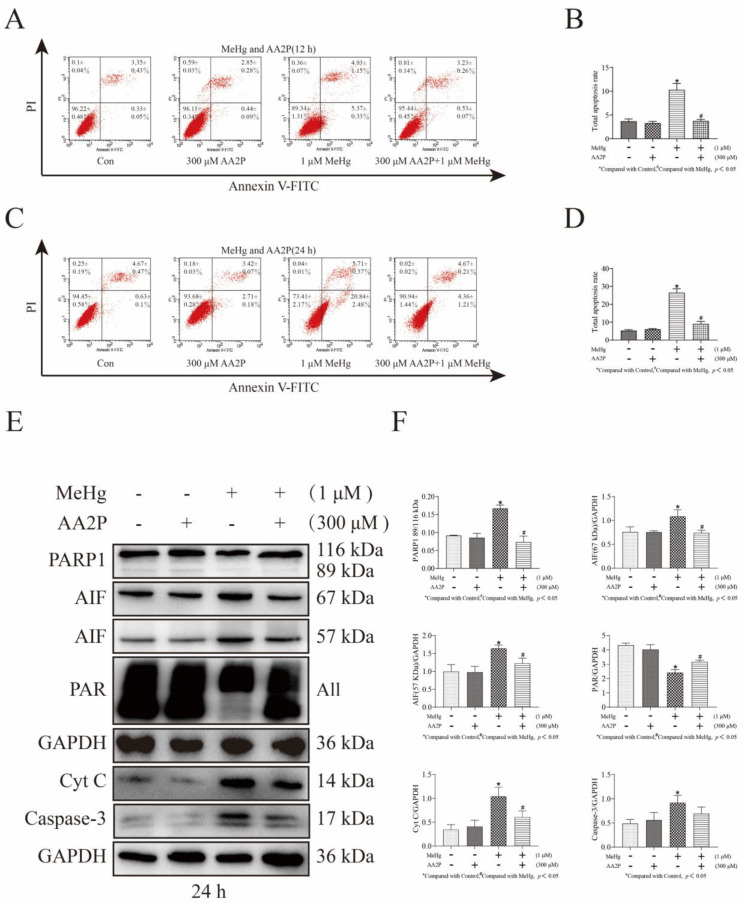
AA2P regulation of the PARP1/AIF and Caspase-3 pathways during MeHg-induced apoptosis of SH-SY5Y cells. (**A**) Flow cytometry detection of apoptosis in SH-SY5Y cells co-treated with 300 μM AA2P and 1 μM MeHg for 12 h. (**B**) GraphPad Prism 8 software analysis of the apoptosis rate of SH-SY5Y cells co-treated with 300 μM AA2P and 1 μM MeHg for 12 h. Statistical significance was determined using ANOVA, *p* < 0.05. (**C**) Flow cytometry detection of apoptosis in SH-SY5Y cells co-treated with 300 μM AA2P and 1 μM MeHg for 24 h. (**D**) GraphPad Prism 8.0.2 software analysis of the apoptosis rate of SH-SY5Y cells co-treated with 300 μM AA2P and 1μM MeHg for 24 h. Statistical significance was determined using ANOVA, *p* < 0.05. (**E**) Western blot analysis of PARP1, PAR, AIF, Cyt C, and cleaved Caspase-3 in SH-SY5Y cells co-treated with 300 μM AA2P and 1 μM MeHg for 24 h. (**F**) The Tanon-2500 fully automated digital gel image analysis system was used to analyze the net optical density of the Western blot strips. All values are represented as means ± SD (*n* = 3).

To demonstrate that AA2P treatment can reduce MeHg-induced apoptosis, we first examined the apoptosis rate of SH-SY5Y cells co-treated with 300 μM AA2P and 1 μM MeHg for 12 and 24 h by flow cytometry. We found that 1 μM MeHg treatment of SHSY5Y cells for 12 h increased the total apoptosis rate of SH-SY5Y cells from 3.68% to 10.3% (*p* < 0.05; Figure 5A,B). MeHg treatment for 24 h increased the total apoptosis rate of SH-SY5Y cells from 5.3% to 26.55% (*p* < 0.05; Figure 5C,D). The 300 μM AA2P and 1 μM MeHg co-treatment of SH-SY5Y cells for 12 h reduced MeHg-induced apoptosis (compared with the MeHg-treated group, the total apoptosis rate decreased from 10.3% to 3.76%, *p* < 0.05; Figure 5A,B). Co-treatment of SH-SY5Y cells with 300 μM AA2P and 1 μM MeHg for 24 h reduced MeHg-induced apoptosis (compared with the MeHg-treated group, the total apoptosis rate decreased from 26.55% to 9.03%, *p* < 0.05; Figure 5C,D). Western blot analysis revealed that the treatment of SH-SY5Y cells with 1 μM MeHg for 24 h downregulated the expression of 116 kDa PARP1 and PAR and upregulated the expression of 67 kDa AIF, 57 kDa AIF, 14 kDa Cyto C, and cleaved Caspase-3 (*p* < 0.05; Figure 5E,F). When SH-SY5Y cells were co-treated with 300 μM AA2P and 1 μM MeHg for 24 h, the expression of 116 kDa PARP1, 116/89 kDa PARP1, and PAR was upregulated, while the expression of 67 kDa, 57 kDa AIF, and 14 kDa Cyto C was downregulated (*p* < 0.05; Figure 5E,F). However, the downregulation of cleaved Caspase-3 expression was not significant.

### 3.6. AA2P Reduces MeHg-Induced DNA Damage and Repair in SH-SY5Y Cells

**Figure 6:**
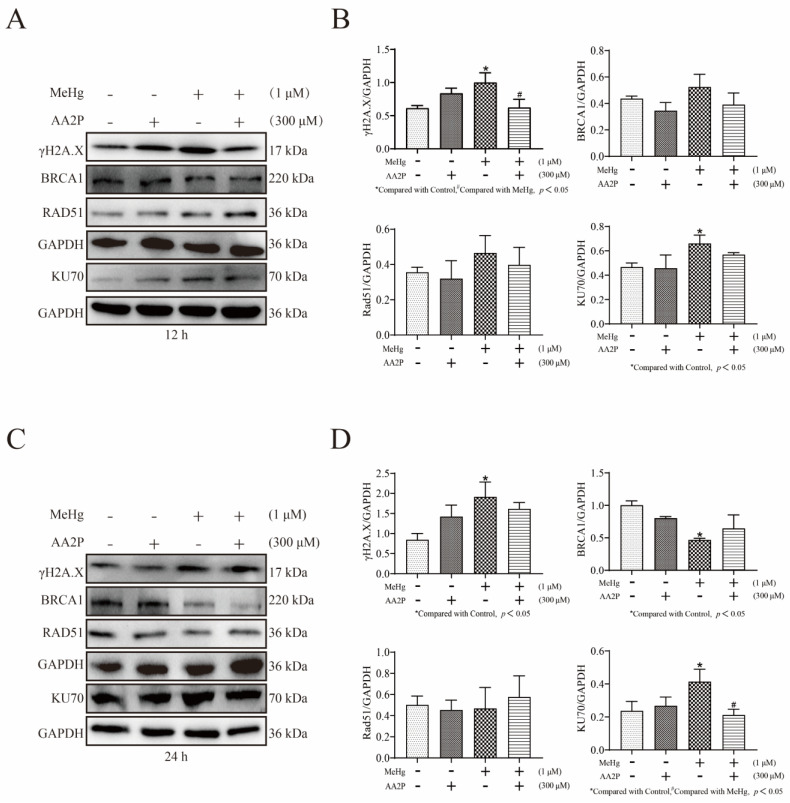
AA2P reduces MeHg-induced DNA damage and NHEJ repair in SH-SY5Y cells. (**A**) Western blot analysis of γH2A.X, BRCA1, RAD51, and KU70 expression in SH-SY5Y cells co-treated with 300 μM AA2P and 1 μM MeHg for 12 h. (**B**) The Tanon-2500 fully automated digital gel image analysis system was used to analyze the net optical density of the Western blot strips. (**C**) Western blot analysis of γH2AX, BRCA1, RAD51, and KU70 expression in SH-SY5Y cells co-treated with 300 μM AA2P and 1 μM MeHg for 24 h. (**D**) The Tanon-2500 fully automated digital gel image analysis system was used to analyze the net optical density of the Western blot strips. All values are represented as means ± SD (*n* = 3).

To investigate the effect of AA2P treatment on MeHg-induced DNA damage and damage repair in SH-SY5Y cells, we selected 300 μM AA2P and 1 μM MeHg for the cotreatment of SH-SY5Y cells for 12 and 24 h. A Western blot analysis showed that 1 μM MeHg treatment of SH-SY5Y cells for 12 and 24 h upregulated the expression of γH2AX (*p* < 0.05; Figure 6A–D). In contrast, 300 μM AA2P + 1 μM MeHg co-treatment significantly downregulated MeHg-induced γH2AX expression (*p* < 0.05; Figure 6A–D). The addition of the antioxidant AA2P reduces cellular damage, but its effect on DNA damage repair is unknown. Therefore, we examined DNA double-strand break repair-related proteins using Western blot analysis and found that MeHg treatment of SH-SY5Y cells at both 12 and 24 h upregulated KU70 expression (*p* < 0.05, Figure 5A–D). MeHg treatment of SH-SY5Y cells for 24 h downregulated the expression of BRCA1 (*p* < 0.05, Figure 5C,D). When SH-SY5Y cells were co-treated with 300 μM AA2P + 1 μM MeHg for 24 h, the expression of the NHEJ repair-related protein KU70 was downregulated (compared with MeHg group, *p* < 0.05; Figure 6 C,D). However, 300 μM AA2P + 1 μM MeHg co-treatment of SH-SY5Y cells for 12 and 24 h did not significantly upregulate BRCA1 and RAD51 expression (Figure 6A–D).

## 4. Discussion

To successfully construct a MeHg-induced cell damage model, we selected SH-SY5Y cells treated with 0–4 μM MeHg for 0–48 h. Cell survival decreased with increasing MeHg concentration and time, and there was a time–dose response relationship. Flow cytometry revealed that MeHg induces apoptosis in SH-SY5Y cells, and Western blot analysis revealed that MeHg induces mitochondria-mediated apoptosis in SH-SY5Y cells in the presence of the PARP1/AIF and Caspase-3 apoptotic pathways. Based on these results, we selected 1 μM MeHg treatment of SH-SY5Y cells for 24 h as a model treatment for cell injury, though we found that MeHg induced ROS accumulation in SH-SY5Y cells as early as 3 h and apoptosis in SH-SY5Y cells at 6 h. These results indicate that MeHg increases ROS accumulation in SH-SY5Y cells in a time-dependent manner and is accompanied by an increase in apoptosis. These results are also consistent with another report that 1 μM MeHg induces oxidative stress and subsequent cell death in SH-SY5Y cells [22]. MeHg was previously shown to increase intracellular ROS [16,17], causing lipid peroxidation and leading to intracellular oxidative stress [30]. Together, these findings indicate that oxidative stress is one of the main mechanisms of MeHg-induced CNS damage [51].

A DNA DSB occurs when both strands of the DNA are cut. DSBs may arise spontaneously under conditions of DNA replication stress due to cellular exposure to exogenous mediators, such as ionizing radiation, or certain exogenous compounds. As DSBs are cytotoxic lesions that threaten genomic integrity and stability, cells are typically repaired by one of two major methods, NHEJ or HR repair of DSBs [52]. NHEJ involves direct ligation of DNA ends and is generally considered error-prone because sequence gain or loss can occur prior to end joining. In the NHEJ pathway, PAR mediates the recruitment of the Ligase IV-XRCC4 complex, aggregates KU70 and KU80 at the DNA break, and forms dimers, and then PARP1 activates and recruits DNA-PKcs, which work together to repair DNA damage [53]. In contrast, HR is more accurate because homologous DNA sequences, usually from a homologous chromosome or sister chromatid, are used as repair templates. Following DNA damage, BRCA1 is recruited to the DSB, where it promotes end resection and recruits the central recombinase RAD51 to initiate HR repair [54]. Among these repair-associated elements, PAR is recognized by several repair mechanisms, such as the BRCA1-BARD1, MRN (MRE11, RAD50, NBS1), and hSSB1-INTS complexes [55]. However, there are still no relevant reports on the repair pathway associated with MeHg-induced DSBs.

As supported by our work, MeHg is known to damage DNA and cause DSBs [23]. We found an increase in the extent of DNA damage in SH-SY5Y cells treated with MeHg for 6–24 h in the alkaline comet assay. It is worth noting that the neutral comet assay is mainly used to detect DNA single-strand damage, while the alkaline comet assay can detect DNA single-strand and double-strand damage. Therefore, to better determine the time point of DSB injury, we detected the expression of the DSB-specific marker γH2A.X in MeHg-treated SH-SY5Y cells using Western blot analysis. Our results revealed that MeHg treatment of SH-SY5Y cells for 12 and 24 h significantly upregulated the expression of γH2AX. This finding indicates that MeHg specifically induces DNA DSBs in SH-SY5Y cells. However, only one study, which was conducted on zebrafish liver, skeletal muscle, and brain, has reported the effect of MeHg on the DNA repair gene RAD51 [56]. The effect of MeHg on the HR-related repair proteins BRCA1 and RAD51 and the NHEJ-related repair protein KU70 in DSB repair pathways has not yet been reported. Our results revealed that MeHg treatment of SH-SY5Ycells for 24 h downregulated the expression of BRCA1 and RAD51 and upregulated that of KU70. Our findings indicate that MeHg could reduce HR repair and increase NHEJ repair in SH-SY5Y cells and that MeHg is a potential genotoxic agent.

PARP1 is an abundant nuclear chromatin-associated protein that exhibits different cellular functions depending on the nature and intensity of extrinsic and intrinsic stress stimuli. On the one hand, PARP1 is highly capable of sensing DNA damage. Once it encounters the free DNA end, PARP1 is catalytically activated and generates PAR chains [57], which can act as a scaffold to recruit various DNA repair proteins and molecules to initiate DNA repair and maintain DNA integrity [58]. On the other hand, hyperactivated PARP1 induces cell death by inducing the nuclear translation of AIF and large DNA fragmentation [59], which is also known as “Parthanatos” [60]. In addition, Caspase-3 can cleave 116 kDa PARP1 into an 89 kDa PARP1 fragment, which acts as a cytoplasmic PAR carrier to induce AIF-mediated apoptosis [61,62]. In our work, we found that MeHg induces mitochondria-mediated apoptosis in SH-SY5Y cells, downregulates the expression of 116 kDa PARP1, upregulates the expression of AIF and Caspase-3, and activates both the caspase cascade and PARP1. These findings are consistent with the reported neurotoxicity demonstrated by MeHg to induce apoptosis in SH-SY5Y cells [40].

MeHg can induce both ROS production and DSB in SH-SY5Y cells, but the relationship between ROS and DNA damage remains unclear. Therefore, we considered reducing MeHg-induced DNA damage and apoptosis caused by using antioxidants to inhibit ROS production. The commonly used antioxidants currently used to inhibit cellular ROS are VC and N-acetylcysteine (NAC). It is well known that MeHg has a high affinity for the anionic form of thiol groups and can react specifically with -SH groups of cellular proteins and non-protein molecules, forming stable complexes with defined stoichiometry [63]. In a previous experiment, NAC was chosen as a co-treatment with MeHg to reduce the toxicity of MeHg; however, NAC is a precursor of glutathione, a sulfhydryl (-SH)-containing antioxidant, thereby affecting the accuracy of the experimental results. Therefore, we chose the antioxidant AA2P to inhibit MeHg-induced ROS in SHSY5Y cells and examined whether AA2P attenuated MeHg-induced DNA damage and apoptosis by alleviating ROS. Our results reveal that low-dose AA2P promotes cell survival, attenuates MeHg-induced ROS production in SH-SY5Y cells, and attenuates MeHg-induced DNA damage and apoptosis. AA2P treatment also upregulates the expression of PARP1 and PAR and downregulates the expression of AIF, Cyt C, and Caspase-3. Together, our results indicate that AA2P can rescue the severe cellular damage caused by MeHg. Our findings are consistent with reports that the addition of AA2P can reduce MeHg-induced cytotoxicity [47,50]. Notably, these studies demonstrated that high doses of VC reduce the viability of cancer cells but not normal cells. High-dose VC also enhances ROS generation and induces apoptosis; therefore, high-dose VC is used clinically to treat tumors [64]. Through our AA2P and MeHg co-treatment experiments, we demonstrate that MeHg-induced DNA damage and apoptosis are mainly caused by ROS. However, AA2P downregulates the expression of the NHEJ repair-related protein KU70, though not the HR repair-related proteins BRCA1 and RAD51. Ultimately, these findings suggest that AA2P alleviates the potential genotoxicity of MeHg.

Currently, the protective effect of antioxidants against MeHg neurotoxicity is mainly attributed to experimental studies, such as our own. MeHg exposure in humans is long-term and in relatively low concentrations, through the ingestion of contaminated fish. Ingestion of contaminated substances is not a conducive method for researching the effect of antioxidants on MeHg in humans. Additionally, although significant progress has been made in understanding the mechanisms of MeHg behind oxidative stress and neurodevelopmental toxicity, the precise molecular targets of MeHg are still not fully understood. The oxidative stress caused by MeHg and its mechanism of action is complex; therefore, further studies are necessary to identify the primary targets of MeHg. Authors should discuss the results and how they can be interpreted from the perspective of previous studies and of the working hypotheses. The findings and their implications should be discussed in the broadest context possible. Future research directions may also be highlighted.

## 5. Conclusions

**Figure 7:**
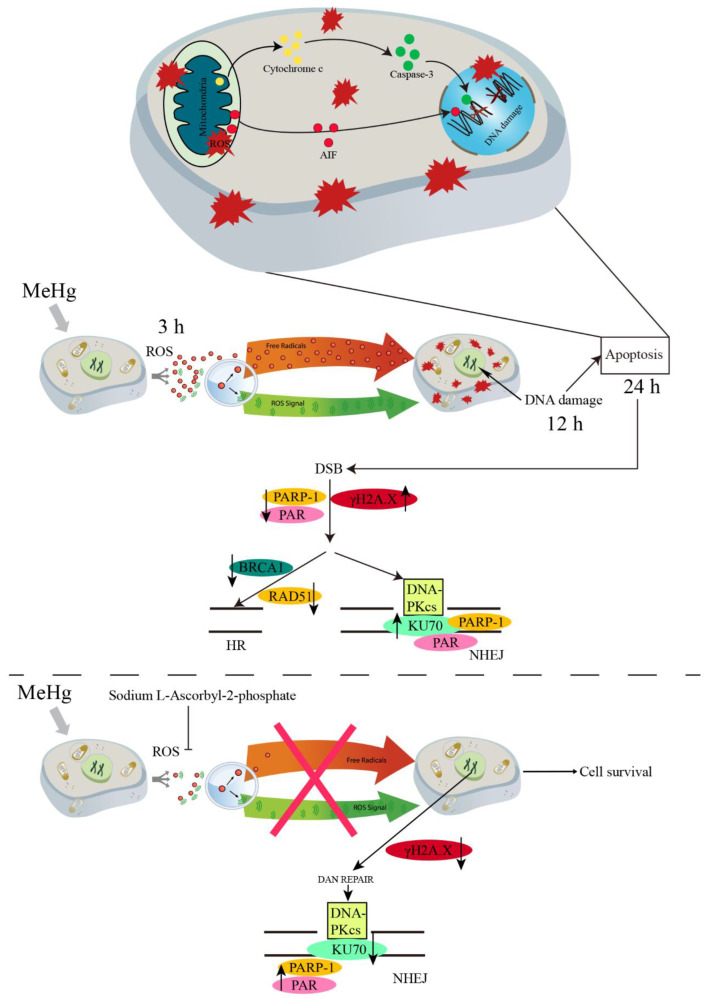
Schematic representation of the effects of MeHg and AA2P on SH-SY5Y cells. AA2P reduces MeHg-induced damage and apoptosis in SH-SY5Y cells by attenuating ROS-mediated DNA damage and downregulating NHEJ repair-related protein KU70.

In this study, we found that MeHg-induced ROS generation in SH-SY5Y cells, DNA damage, and mitochondria-mediated apoptosis. After 24 h, MeHg-treated SH-SY5Y cells showed increased NHEJ repair and decreased HR repair. However, the antioxidant AA2P reduced MeHg-induced intracellular ROS production and alleviated MeHg-induced cell damage and apoptosis (Figure 7). Ultimately, it can be speculated that MeHg-induced DNA damage and apoptosis are mainly caused by ROS and that the generation of ROS precedes DNA damage and apoptosis. However, it is worth noting that AA2P reduces NHEJ repair, indicating that AA2P can reduce MeHg-induced error-prone DNA repair.

## Data Availability

All data are included within the article.
